# Shigella sonnei Does Not Use Amoebae as Protective Hosts

**DOI:** 10.1128/AEM.02679-17

**Published:** 2018-04-16

**Authors:** Jayne Watson, Claire Jenkins, Abigail Clements

**Affiliations:** a MRC Centre for Molecular Bacteriology and Infection, Department of Life Sciences, Imperial College London, London, United Kingdom; b Gastrointestinal Bacteria Reference Unit, Public Health England, Colindale, London, United Kingdom; University of Minnesota

**Keywords:** amoeba, Shigella sonnei, intracellular survival

## Abstract

Shigella flexneri and Shigella sonnei bacteria cause the majority of all shigellosis cases worldwide. However, their distributions differ, with S. sonnei predominating in middle- and high-income countries and S. flexneri predominating in low-income countries. One proposed explanation for the continued range expansion of S. sonnei is that it can survive in amoebae, which could provide a protective environment for the bacteria. In this study, we demonstrate that while both S. sonnei and S. flexneri can survive coculture with the free-living amoebae Acanthamoebae castellanii, bacterial growth is predominantly extracellular. All isolates of Shigella were degraded following phagocytosis by A. castellanii, unlike those of Legionella pneumophila, which can replicate intracellularly. Our data suggest that S. sonnei is not able to use amoebae as a protective host to enhance environmental survival. Therefore, alternative explanations for S. sonnei emergence need to be considered.

**IMPORTANCE** The distribution of Shigella species closely mirrors a country's socioeconomic conditions. With the transition of many populous nations from low- to middle-income countries, S. sonnei infections have emerged as a major public health issue. Understanding why S. sonnei infections are resistant to improvements in living conditions is key to developing methods to reduce exposure to this pathogen. We show that free-living amoebae are not likely to be environmental hosts of S. sonnei, as all Shigella strains tested were phagocytosed and degraded by amoebae. Therefore, alternative scenarios are required to explain the emergence and persistence of S. sonnei infections.

## INTRODUCTION

Shigella is a genus of Gram-negative enteric pathogens comprised of four species. All spe*c*ies can cause severe diarrhea, and Shigella is estimated to cause 165 million infections and 120,000 deaths annually, accounting for 10% of deaths due to diarrheal disease worldwide (1, 2). Shigella flexneri and Shigella sonnei cause the majority of infections, but the ratio of species dominance is highly dependent on the socioeconomic conditions of the area. In countries with a low per capita income, including those of sub-Saharan Africa and some countries in Asia, S. flexneri is the dominant cause of shigellosis, responsible for over 60% of infections. However, in areas with a high human development index, such as Europe and North America, S. sonnei causes around 80% of shigellosis cases (3). Transitioning countries currently undergoing socioeconomic improvements are experiencing a shift in the dominant species causing infections, from S. flexneri to S. sonnei. From 2001 to 2008 the prevalence of S. flexneri in Bangladesh decreased from 65.7% to 47%, while the prevalence of S. sonnei increased from 7.2% to 25% (4). During this time, Bangladesh underwent significant improvements in the nutritional status of children, health care, and water sanitation (5, 6). Other countries, such as China, Vietnam, and Brazil, have experienced a similar trend (7–9).

The reason for the rising dominance of S. sonnei in areas where the S. flexneri infection rate is decreasing is unclear. One hypothesis is that S. sonnei can use amoebae as environmental hosts to protect it from water sanitation measures that are implemented in transitional countries (10). Amoebae are free-living organisms found in a variety of water sources, such as swimming pools and lakes, as well as in soil and dust. Importantly, they have even been found in chlorinated public water sources in developed countries (11). They are able to tolerate harsh and changing conditions, making them a good host for a variety of bacteria (12). Legionella pneumophila is the most well-known bacterium known to utilize amoebae as protective hosts, but Campylobacter jejuni, Salmonella enterica serovar Typhimurium, and Vibrio cholerae have also been shown to survive intracellularly in amoebae (13–15, 16). However, some bacteria which were initially described as surviving in amoebae have later been shown to grow extracellularly, potentially through saprophytic growth on dead amoebae or amoeba waste (17–20).

Previous work has suggested that S. sonnei can survive in amoebae for extended periods of time. S. sonnei, Shigella dysenteriae, and S. flexneri were all found to be phagocytosed by Acanthamoebae castellanii; however, only S. sonnei and S. dysenteriae appeared to survive and replicate in the cytosol (21).

Here, we explore the hypothesis that amoebae can act as an environmental reservoir for S. sonnei. Although S. sonnei is phagocytosed by amoebae, we found no evidence that S. sonnei is able to survive and replicate in the cytosol of A. castellanii.

## RESULTS

### Shigella cells survive extended coculture with amoebae.

**FIG 1:**
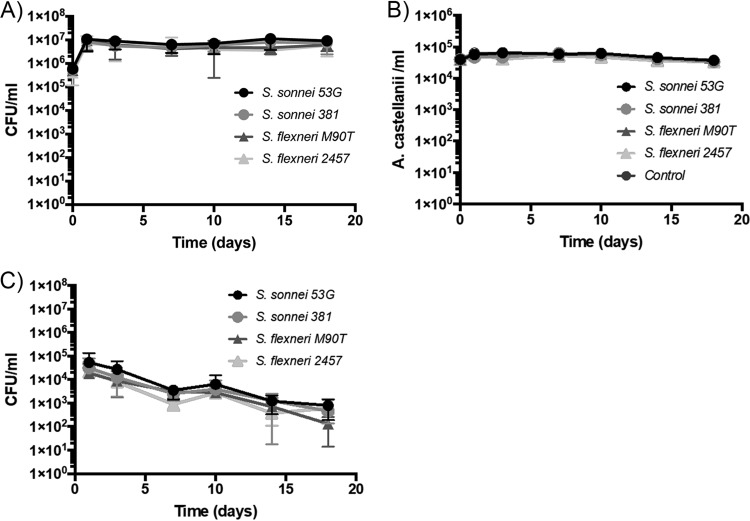
All Shigella strains survive extended coculture with A. castellanii. Shigella strains were cocultured with A. castellanii for 18 days in low-nutrient medium supplemented with heat-killed E. coli cells. At the indicated time points, the following measurements were made: (A) total bacteria determined by colony counts; (B) number of amoebae quantified by microscopy; (C) number of gentamicin-protected intracellular bacteria determined by colony counts.

Consistent with previous research, we observed that strains of S. sonnei and S. flexneri were able to survive in coculture with A. castellanii over 18 days at 22°C. We used two S. flexneri serotypes (strain M90T, serotype 5a, and strain 2457T, serotype 2a) and two S. sonnei isolates (the commonly used 53G strain and a recent clinical isolate, H140860381, here referred to as 381). All strains remained culturable at 10^6^ to 10^7^ CFU/ml over the 18 days (Fig. 1A). The amoebae were also maintained at ca. 5 × 10^4^ cells/ml throughout this time period (Fig. 1B). These data indicate that Shigella species can survive extended coculture in the presence of amoebae but give no information as to whether the bacteria are residing within amoebae and potentially utilizing the amoebae as an environmental reservoir.

We determined the intracellular bacterial numbers by taking samples at the indicated time points, treating with gentamicin to kill extracellular bacteria, and lysing the amoebae prior to CFU determination (Fig. 1C). This analysis revealed that all bacterial strains could be recovered intracellularly at all time points. However, fewer intracellular bacteria were recovered at the later time points. No difference was observed in intracellular bacterial numbers between the S. flexneri and S. sonnei strains at any time point.

The intracellular bacteria observed in this assay could be recently phagocytosed bacteria that had not yet been degraded, or bacteria that had established an intracellular niche and were surviving and/or replicating. We sought to examine these possibilities further.

### All Shigella strains are phagocytosed by A. castellanii.

**FIG 2:**
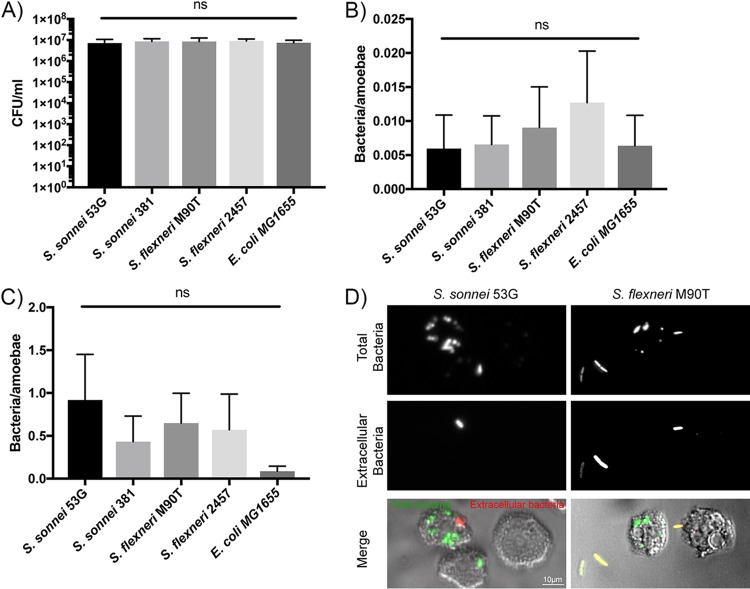
Shigella and E. coli show similar levels of amoeba association and phagocytosis at 22°C. A. castellanii was incubated with the indicated bacterial strains for 1 h, washed, and then either (A) followed by determination of cell-associated bacteria or (B and C) treated with gentamicin for 1 h to kill extracellular bacteria to determine the number of phagocytosed bacteria. (A and B) Experiments were conducted in low-nutrient medium or (C) high-nutrient medium. One-way analysis of variance (ANOVA) indicated no statistically significant differences between any bacterial strains. Mean and a standard deviation of 5 repeats are shown. (D) S. sonnei 53G and S. flexneri M90T strains expressing GFP were incubated with A. castellanii and washed, and extracellular bacteria were detected with specific antibodies prior to visualization. Individual fluorescence channels for total and extracellular bacteria are shown in the top panels. The bottom panel represents merged transmitted-light images (to visualize the amoebae) and fluorescence images (green, total bacteria; red, extracellular bacteria).

To determine the efficiency of phagocytosis of Shigella strains, amoebae incubated in low-nutrient medium (Page’s modified Neff’s amoeba saline [PAS]) at 22°C were allowed to phagocytose bacteria for 1 h, which was followed by 1 h gentamicin treatment to kill extracellular bacteria. The numbers of total bacteria (prior to gentamicin treatment) were similar for all strains and indicated that, on average, 5 × 10^6^ CFU/ml, or approximately 50 bacteria/amoeba, were present (Fig. 2A). Following gentamicin treatment, recoverable CFU decreased by 4 log, indicating that the majority of the bacteria were extracellular or were rapidly degraded by the amoebae during the gentamicin incubation (Fig. 2B). There was no significant difference in the numbers of amoeba-associated bacteria or phagocytosed bacteria between nonpathogenic Escherichia coli strain MG1655 and the Shigella strains, or between the Shigella species.

We repeated the experiments in high-nutrient medium (peptone-yeast-glucose medium [PYG]) to increase the rate of phagocytosis by amoebae. As anticipated, significantly higher numbers of phagocytosed bacteria could be enumerated than in low-nutrient medium (Fig. 2C). On average, there were 0.5 intracellular bacteria/amoeba. However, we again saw no difference between the phagocytosis rates for S. flexneri or S. sonnei strains. All Shigella strains showed a small, nonsignificant trend of increased phagocytosis by amoebae compared to that for nonpathogenic E. coli. Microscopic analysis of differentially stained bacteria confirmed the presence of intracellular bacteria for both S. sonnei and S. flexneri (Fig. 2D). Extracellular bacteria can be seen adhered to the plastic, rather than to the amoebae, suggesting that the amoebae efficiently phagocytose all bacteria contacted.

### Shigella does not survive intracellularly in A. castellanii.

**FIG 3:**
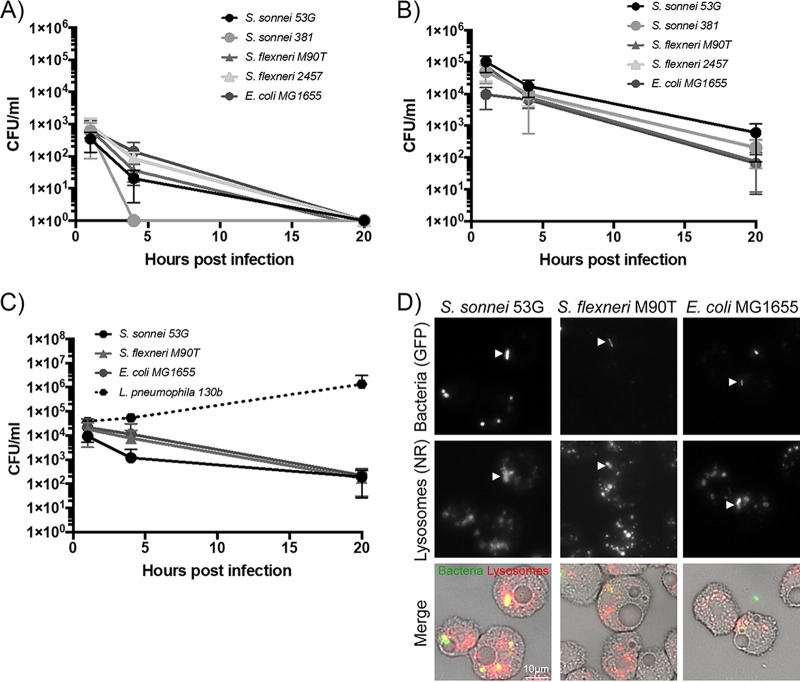
Shigella cells are degraded by amoebae while Legionella pneumophila cells are able to replicate within amoebae. (A to C) The indicated bacterial strains were incubated with A. castellanii for 1 h and then washed and treated with gentamicin for 1 h to kill extracellular bacteria, and the number of bacteria able to survive amoebae degradation was determined after 1 h, 4 h, and 20 h of gentamicin treatment. Experiments were conducted in low-nutrient medium at 22°C (A), high-nutrient medium at 22°C (B), and high-nutrient medium at 30°C (C). Mean and a standard deviation of 3 to 5 repeats are shown. (D) Amoebae stained with neutral red (NR), which preferentially accumulates in lysosomes, were imaged immediately following phagocytosis of the indicated GFP-expressing bacteria. Individual fluorescence channels for bacteria and lysosomes are shown in the top panels. The bottom panel represents merged transmitted-light images (to visualize the amoebae) and fluorescence images (green, bacteria; red, NR lysosomes). Arrows indicate bacteria that are being infiltrated with neutral red dye, indicating lysosomal digestion by the amoebae.

We tested the intracellular survival of Shigella by CFU determination at 1 h, 4 h, and 20 h. Cell counts for all strains decreased over this time frame similarly to those of the negative control of nonpathogenic E. coli, indicating they were efficiently digested by A. castellanii. The same trend of reduced intracellular numbers over time was observed in both high- (Fig. 3A) and low-nutrient media (Fig. 3B), with a 2-log decrease between 1 h and 20 h. Due to the low number of phagocytosed bacteria in low-nutrient medium, by 20 h all strains were below the limit of detection, unlike in high-nutrient medium, where approximately 1,000 bacteria/sample were still recoverable.

To confirm that the amoebae were capable of facilitating intracellular growth, L. pneumophila was used as a positive control. These experiments were conducted at 30°C, as this is the optimal temperature for L. pneumophila growth (Fig. 3C). As expected, the numbers of culturable wild-type L. pneumophila cells increased over the 20 h of incubation, whereas cell counts for all Shigella strains again decreased, in line with those of nonpathogenic E. coli.

To investigate the intracellular fate of bacteria, we observed the association of bacteria with amoebae stained with neutral red (Fig. 3D). Neutral red preferentially accumulates in lysosomes, due to their relative acidity (22, 23). S. flexneri, S. sonnei, and E. coli were all observed to have neutral red structures accumulating around the intracellular bacteria and to be infiltrated with neutral red, suggesting they were being digested by the amoebae (24). This provides a visual confirmation of the intracellular killing observed in the preceding assays by bacterial enumeration.

### The T3SS does not alter Shigella interaction with amoebae.

Considering the importance of the type 3 secretion system (T3SS) for Shigella virulence, the intracellular survival assays were repeated at 37°C, the temperature at which the T3SS is active and effector proteins are translocated (25). Shigella bacteria induce different T3SS-dependent outcomes depending on the cell type infected; in epithelial cells, vacuolar escape and intracellular replication, and in macrophages, vacuolar escape and pyroptosis.

**FIG 4:**
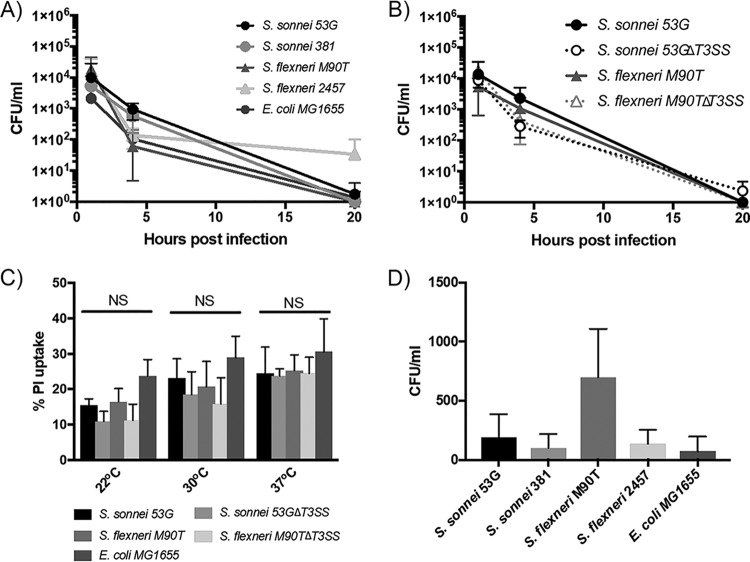
The T3SS does not enhance Shigella survival in amoebae. (A) Following phagocytosis, the number of bacteria able to survive degradation by amoebae was determined after 1 h, 4 h, and 20 h of gentamicin treatment during incubation at 37°C to activate the T3SS. (B) The intracellular survival was also determined for S. flexneri M90T and S. sonnei 53G T3SS mutants and compared to those of parental strains. (C) Propidium iodide uptake was used to measure membrane integrity in amoebae exposed to the indicated strains for 3 h. All values are a percentage of the maximum PI uptake calculated for amoebae exposed to 0.25% sodium deoxycholate for 10 min. (D) The indicated bacterial strains were incubated with A. castellanii for 1 h and then washed and treated with gentamicin for 1 h to kill extracellular bacteria. Fresh medium without gentamicin was then added for a further 3 h, after which time the supernatant was harvested and the number of released bacteria determined by colony counting. For all assays, two-way ANOVA indicated no significant differences between any strains at any time point. Mean and a standard deviation of 3 repeats are shown.

If the T3SS facilitated intracellular survival within amoebae, we would expect to see increased intracellular bacterial counts at 4 h and 20 h during incubation at 37°C. Instead, we saw a decrease in viable intracellular bacteria numbers, similar to those at 22°C and 30°C, suggesting that an active T3SS could not facilitate intracellular survival in amoebae (Fig. 4A). To further investigate the involvement of the T3SS, the intracellular survival of T3SS mutants was determined. Again, no difference in intracellular survival between wild-type and T3SS mutants was found (Fig. 4B), indicating that the T3SS was not altering the interaction of Shigella with amoebae.

It was previously reported that S. flexneri used its T3SS to kill amoebae (21, 26). Having not seen an effect of the T3SS (Fig. 4A and B), or significant amoeba death upon long-term exposure to S. flexneri (Fig. 1C), we decided to investigate amoeba cell death further by using a propidium iodide (PI) assay to measure the membrane integrity of the amoebae (Fig. 4C). At all temperatures analyzed, there were no significant differences in PI levels between amoebae infected with the negative controls (E. coli MG1655 and Shigella T3SS mutants) and any of the wild-type Shigella strains. Therefore, in our assays neither S. flexneri or S. sonnei were able to induce cell death in amoebae.

### S. sonnei is not released by amoebae.

V. cholerae was recently shown to resist intracellular killing by A. castellanii, and at low frequency it can be released intact by the amoebae (16). While we have seen no evidence of Shigella resisting intracellular killing, we questioned whether a small number of bacteria were being released from the amoebae. Following killing of extracellular bacteria with gentamicin treatment, fresh medium with no gentamicin was added to cells, and the cell supernatant was harvested after 4 h (Fig. 4D). Low numbers of Shigella were recovered from the supernatant, and these could potentially be a source of Shigella for infection. However, there was no significant difference in bacterial release between S. sonnei and S. flexneri strains, and indeed, no significantly increased numbers of bacteria were released compared to those for nonpathogenic E. coli, indicating this is not a Shigella- or S. sonnei-specific mechanism for dispersal.

### Intracellular Shigella bacteria are not more infectious.

**FIG 5:**
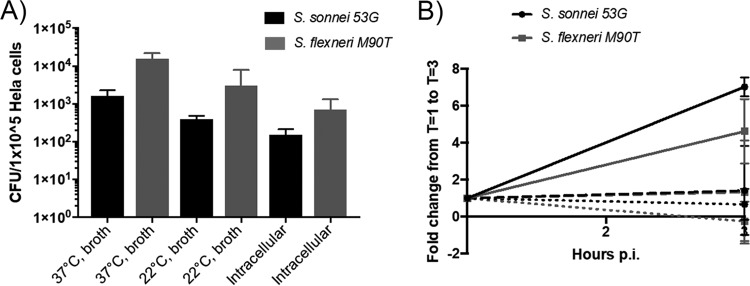
Intracellular bacteria are not hyperinfectious for epithelial cells. (A) Bacteria harvested from amoebae following 1 h of gentamicin treatment were used to directly infect epithelial cells, in parallel with standard log-phase bacteria grown in TSB at 37°C or 22°C. Following a 30 min infection and 1 h of gentamicin treatment, intracellular bacteria were released, and invasive bacteria was enumerated by colony counting. (B) The intracellular survival of these bacteria was measured after 3 h of gentamicin treatment. The fold change from 1 h to 3 h postinfection was calculated. Solid lines indicate bacteria grown in TSB at 37°C, dashed lines indicate bacteria grown in TSB at 22°C, and dotted lines indicate bacteria harvested from amoebae.

Amoebae have been proposed to act as “training grounds” for intracellular pathogens, adapting them to an intracellular lifestyle (27). While this is considered a long-term adaptation, we questioned whether it facilitated short-term infectivity as well. We therefore tested whether bacteria harvested from amoebae were more proficient at invading or replicating within mammalian cells. We found the bacteria harvested from amoebae were less able to invade and survive in mammalian cells (Fig. 5A and B). These findings support the previous conclusion that Shigella bacteria are being degraded by the amoebae, rather than adapting and surviving.

## DISCUSSION

The frequency of S. sonnei isolation directly correlates with per capita gross domestic product (GDP) (28). The underlying reason(s) for this association is not understood, although a number of hypotheses have been proposed, one of which is that S. sonnei uses amoebae as a protective host (10). We show here that S. sonnei has no survival advantage in amoebae compared to S. flexneri, or indeed, compared to nonpathogenic E. coli. Both Shigella species were able to survive in long term coculture assays in low-nutrient medium suggesting that, like Listeria monocytogenes, Shigella species can utilize amoeba debris for nutritional requirements (17). However, this offers no explanation for why S. flexneri, but not S. sonnei, levels of infection are reduced in areas where living conditions and water sanitation are improved.

Having disproven one hypothesis explaining the emergence of S. sonnei, it remains to experimentally test additional hypotheses. One popular suggestion is that exposure to unsanitized water in developing countries can result in Plesiomonas shigelloides infection and hence in natural immunity against *S. sonnei. P. shigelloides* serotype O17 has a lipopolysaccharide O-antigen identical to that of S. sonnei (10). People living in areas with good water sanitation would therefore have reduced exposure to P. shigelloides, and hence reduced cross-protection against S. sonnei. This hypothesis is difficult to prove without widespread serological data from countries with high S. flexneri versus high S. sonnei infection rates. However, it also suggests additional differences regarding the transmission of S. sonnei, as the reduced exposure to P. shigellelloides and S. flexneri through improved water quality does not extend to S. sonnei. This could be explained by the suggestion that S. sonnei is spread directly from person to person or that S. sonnei has an increased ability to acquire antibiotic resistance. Acquisition of antimicrobial resistance has clearly aided the spread and establishment of particular S. sonnei isolates (29). However, epidemiological data indicate that S. flexneri and S. sonnei isolates have similar resistance profiles (30, 31), suggesting antibiotic resistance alone does not explain the altered transmission.

S. sonnei possesses multiple antibacterial mechanisms. The majority of clinical isolates produce colicins (29, 32, 33), which are effective against a narrow phylogenetic range of bacteria. While S. flexneri cells are also reported to produce bacteriocins (34), there are few studies indicating the prevalence or identity of bacteriocins in S. flexneri clinical isolates. S. sonnei has also recently been shown to have a functional type 6 secretion system (T6SS), which provides a niche-specific competitive advantage for S. sonnei over S. flexneri (35). Therefore, the success of S. sonnei may be explained by a combination of these factors potentially altering colonization dynamics and facilitating person-to-person spread.

We have demonstrated that amoebae are not a protective host for S. sonnei and that alternative explanations for the rising rates of S. sonnei infection in transitional countries require further investigation. Now that it is well established that S. sonnei possesses unique pathogenic traits (35–37), considerable work is required to understand the differences in virulence and transmission of S. sonnei in comparison to those of S. flexneri.

## MATERIALS AND METHODS

### Bacterial strains and growth.

Isolates of Shigella (Table 1) were plated on trypticase soya agar (TSA) + 0.01% Congo red to identify those with a large virulence plasmid (LVP) (38). Colonies were inoculated in trypticase soya broth (TSB) and incubated overnight at 37°C and 200 rpm. The overnight culture was diluted 1:100 in TSB and incubated until an optical density at 600 nm (OD_600_) of 0.5 was reached. Bacteria were washed in phosphate-buffered saline (PBS), resuspended in the appropriate medium, and added to cells.

**TABLE 1:** Bacterial strains

Strain	Details	Source and/or reference
S. sonnei 53G	Clinical isolate	36
S. sonnei 381	Clinical isolate H140860381	C. Jenkins, PHE^*b*^
S. flexneri M90T	Serotype 5a	37
S. flexneri 2457T	Serotype 2a	38
S. flexneri M90TΔT3SS	*mxiD* replaced with *aphA-3*, conferring kanamycin resistance	39
S. sonnei 53GΔT3SS	*mxiD* replaced with *aphA-3*, conferring kanamycin resistance	This study
S. sonnei 53G/GFP	Expresses GFP^*a*^ from pUltraGFP-GM	This study, 42
S. flexneri M90T/GFP	Expresses GFP from pUltraGFP-GM	This study
L. pneumophila 130b	Serotype O1; clinical isolate	ATCC BAA-74 (41)

a GFP, green fluorescent protein.

b PHE, Public Health England.

Legionella cells were plated on buffered charcoal-yeast extract (CYE) agar plates for 3 days at 37°C. Bacterial colonies were diluted to OD_600_ = 0.1 in ACES [*N*-(2-acetamido)-2-aminoethanesulfonic acid] yeast extract (AYE) broth and incubated for 21 h at 37°C and 200 rpm.

### Cell culture.

Acanthamoeba castellanii cells (a kind gift from C. Buchrieser, Institut Pasteur) were cultured in ATCC 712 peptone-yeast-glucose medium [PYG] medium 2% protease peptone, 0.1% yeast extract, 0.1 M glucose, 4 mM MgSO_4_·47H_2_O, 0.4 mM CaCl_2_, 0.05 mM Fe(NH_4_)_2_(SO_4_)_2_·6H_2_O, 2.5 mM KH_2_PO_4_, 2.5 mM Na_2_HPO_4_·7H_2_O, and 0.01% Na citrate·2H_2_O) in tissue culture flasks at 22°C. During infections, amoebae were seeded in PAS medium (2 mM NaCl_2_, 16 μM MgSO_4_·7H_2_O, 26 μM CaCl2·2H_2_O, 1 mM Na2HPO4, and 1 mM KH_2_PO_4_) or PYG.

HeLa cells were maintained and seeded in Dulbecco's modified Eagle medium (DMEM), 1,000 mg/ml glucose, supplemented with 10% fetal bovine serum (FBS) and 5% Glutamax and incubated at 37°C and 5% CO_2_.

### Mutagenesis.

The T3SS mutant was created by deletion of *mxiD* using lambda red recombination (39, 40). A DNA fragment was created by amplifying the kanamycin gene from pKD4 (with primer pair 5′-tgtgtaggctggagctgcttc-3′ and 5′-catatgaatatcctccttag-3′), 500 bp upstream of *mxiD* (with primer pair 5′-tagggataacagggtaatcagtggtgctctagtagagc-3′ and 5′-gaagcagctccagcctacacaggtaacaatacaatcaagag-3′) and 500 bp downstream of *mxiD* (with primer pair 5′-ctaaggaggatattcatatgggaagacgaaaaatcattgg-3′ and 5′-tagggataacagggtaatcgacttgttcagcagtaagatc-3′), followed by overlapping PCR (I-SceI sites are underlined). This fragment was cloned into pGEM T-Easy and transformed into S. sonnei 53G alongside pACBSR. *mxiD* was then replaced with the kanamycin resistance gene with the help of lambda red recombinase and I-SceI induced from pACBSR.

### Amoeba coculture.

A. castellanii was seeded at 10^6^ cells/flask in T75 tissue culture flasks in PAS medium. Amoebae were infected with Shigella at a multiplicity of infection (MOI) of 10. Every other day 10^7^ heat-killed (100°C for 20 min) E. coli DH5α was added to flasks to prevent amoebae starvation. At time points indicated, 1 ml of culture was removed. Ten microliters was used to count amoebae on a hemocytometer. Twenty microliters was used to perform serial dilutions to calculate coculture CFU. Remaining sample was washed with PAS and treated with 150 μg/ml gentamicin for 1 h. Amoebae were washed again with PAS and lysed with 0.25% sodium deoxycholate. Serial dilutions were performed to calculate intracellular CFU.

### Infection of amoebae.

A. castellanii was seeded at 1 × 10^5^/ml in 24-well plates and infected with an MOI of 100. To synchronize infection, plates were centrifuged at 600 × *g* for 10 min and then incubated for 50 min at 22°C or 37°C. Cells were washed with PAS, and 150 μg/ml gentamicin in PAS/PYG was then added to cells for 1 h. If required, medium was replaced with 20 μg/ml gentamicin in PAS/PYG for a further 3 h or 19 h. At the time points indicated, cells were washed with PAS and lysed with 0.25% sodium deoxycholate for 10 min. Serial dilutions were carried out and plated to calculate intracellular CFU. Experiments using L. pneumophila were performed at 30°C in PYG, and amoebae were infected with L. pneumophila at an MOI of 1. To enumerate intracellular CFU in these experiments, amoebae were washed in PAS, detached from the well, and lysed by vortexing for 10 s. Serial dilutions were performed and plated on appropriate agar. All other experimental steps were as above.

### Fluorescence microscopy.

Amoebae seeded on 4-well μ-slides (Ibidi) were infected as above with GFP (green fluorescent protein)-expressing S. sonnei 53G and GFP-expressing S. flexneri M90T. Amoebae were then washed with low-fluorescence (LF) medium (41) and placed on ice, and extracellular bacteria were detected with sera against S. sonnei (Remel agglutinating sera) or S. flexneri 5a (Public Health England [PHE]) in 2% BSA in LF medium for 30 min. Amoebae were washed with ice-cold LF medium, followed by anti-rabbit Cy3 (Jackson ImmunoResearch) in 2% BSA in PAS for 30 min. Amoebae were washed with LF medium before being overlaid with agarose and immediately imaged on a Zeiss Axio Observer inverted microscope.

For neutral red staining, amoebae were incubated in 125 μM neutral red in LF medium for 20 min at room temperature (RT). Amoebae were washed in LF medium before addition of bacteria harboring GFP and centrifuged briefly (2 min, 1,000 × *g*). Amoebae were overlaid with agarose and immediately imaged on a Zeiss Axio Observer inverted microscope.

### PI assay.

A. castellanii was seeded as described previously in 24-well plates. Immediately prior to infection, medium was replaced with 5 μM propidium iodide in PAS. Infection was carried out as described, and cells were incubated at 22°C, 30°C, or 37°C. At 1 h postinfection, gentamicin was added directly to wells to a final concentration of 150 μg/ml for 3 h. For 100% membrane permeabilization control, sodium deoxycholate was added to wells at a final concentration of 0.25% for 10 min. Fluorescence was measured at 530/620 nm on a FLUOstar Omega microplate reader (BMG Labtech).

### HeLa cell infection.

HeLa cells were seeded in 96-well plates at 1 × 10^5^ cells/ml 24 h prior to infection. A. castellanii was seeded at 10^7^ cells/flask in T75 flasks in PAS medium, infected with Shigella at an MOI of 100, and incubated at 22°C. After 24 h, amoebae were detached, centrifuged at 500 × *g* for 5 min, and resuspended in 150 μg/ml gentamicin in PAS for 1 h. Amoebae were then washed with PAS and lysed with 0.25% sodium deoxycholate. Released bacteria were centrifuged, washed, and resuspended in DMEM. Broth-cultured Shigella cells were prepared as described above and resuspended in DMEM. Prior to infection, medium was replaced with serum-free DMEM, and HeLa cells then infected at an MOI of 100 with Shigella cells released from amoebae or grown in broth at 37°C or 22°C. Cells were centrifuged at 600 × *g* for 10 min and incubated for 30 min at 37°C and 5% CO_2_. Medium was replaced with 150 μg/ml gentamicin in supplemented DMEM and incubated for a further 1 h or 3 h. At these time points, cells were washed with PBS and lysed with 0.5% Triton X-100. Serial dilutions were performed and plated to calculate intracellular CFU.

## References

[1] Lima IF, Havt A, Lima AA 2015 Update on molecular epidemiology of *Shigella* infection. Curr Opin Gastrenterol 31:30–37. doi:10.1097/MOG.0000000000000136. 25394237

[2] Lozano R, Naghavi M, Foreman K, Lim S, Shibuya K, Aboyans V, Abraham J, Adair T, Aggarwal R, Ahn SY, Alvarado M, Anderson HR, Anderson LM, Andrews KG, Atkinson C, Baddour LM, Barker-Collo S, Bartels DH, Bell ML, Benjamin EJ, Bennett D, Bhalla K, Bikbov B, Bin Abdulhak A, Birbeck G, Blyth F, Bolliger I, Boufous S, Bucello C, Burch M, Burney P, Carapetis J, Chen H, Chou D, Chugh SS, Coffeng LE, Colan SD, Colquhoun S, Colson KE, Condon J, Connor MD, Cooper LT, Corriere M, Cortinovis M, de Vaccaro KC, Couser W, Cowie BC, Criqui MH, Cross M, Dabhadkar KC, 2012 Global and regional mortality from 235 causes of death for 20 age groups in 1990 and 2010: a systematic analysis for the Global Burden of Disease Study 2010. Lancet 380:2095–2128. doi:10.1016/S0140-6736(12)61728-0. 23245604 PMC10790329

[3] The HC, Thanh DP, Holt KE, Thomson NR, Baker S 2016 The genomic signatures of *Shigella* evolution, adaptation and geographical spread. Nat Rev Microbiol 14:235–250. doi:10.1038/nrmicro.2016.10. 26923111

[4] Ud-Din AI, Wahid SU, Latif HA, Shahnaij M, Akter M, Azmi IJ, Hasan TN, Ahmed D, Hossain MA, Faruque AS, Faruque SM, Talukder KA 2013 Changing trends in the prevalence of *Shigella* species: emergence of multi-drug resistant *Shigella sonnei* biotype g in Bangladesh. PLoS One 8:e82601. doi:10.1371/journal.pone.0082601. 24367527 PMC3867351

[5] Montgomery MA, Elimelech M 2007 Water and sanitation in developing countries: including health in the equation. Environ Sci Technol 41:17–24. doi:10.1021/es072435t. 17265923

[6] Faruque AS, Ahmed AM, Ahmed T, Islam MM, Hossain MI, Roy SK, Alam N, Kabir I, Sack DA 2008 Nutrition: basis for healthy children and mothers in Bangladesh. J Health Popul Nutr 26:325–339. 18831228 10.3329/jhpn.v26i3.1899 PMC2740711

[7] Vinh H, Nhu NT, Nga TV, Duy PT, Campbell JI, Hoang NV, Boni MF, My PV, Parry C, Nga TT, Van Minh P, Thuy CT, Diep TS, Phuong le T, Chinh MT, Loan HT, Tham NT, Lanh MN, Mong BL, Anh VT, Bay PV, Chau NV, Farrar J, Baker S 2009 A changing picture of shigellosis in southern Vietnam: shifting species dominance, antimicrobial susceptibility and clinical presentation. BMC Infect Dis 9:204. doi:10.1186/1471-2334-9-204. 20003464 PMC2803792

[8] Sousa MA, Mendes EN, Collares GB, Peret-Filho LA, Penna FJ, Magalhaes PP 2013 *Shigella* in Brazilian children with acute diarrhoea: prevalence, antimicrobial resistance and virulence genes. Memorias do Instituto Oswaldo Cruz 108:30–35. doi:10.1590/S0074-02762013000100005. 23440111 PMC3974317

[9] Mao Y, Cui E, Bao C, Liu Z, Chen S, Zhang J, Wang H, Zhang C, Zou J, Klena JD, Zhu B, Qu F, Wang Z 2013 Changing trends and serotype distribution of *Shigella* species in Beijing from 1994 to 2010. Gut Pathogens 5:21. doi:10.1186/1757-4749-5-21. 23919811 PMC3750644

[10] Thompson CN, Duy PT, Baker S 2015 The rising dominance of *Shigella sonnei*: an intercontinental shift in the etiology of bacillary dysentery. PLoS Negl Trop Dis 9:e0003708. doi:10.1371/journal.pntd.0003708. 26068698 PMC4466244

[11] Wang H, Edwards M, Falkinham JO III, Pruden A 2012 Molecular survey of the occurrence of *Legionella* spp., *Mycobacterium* spp., *Pseudomonas aeruginosa*, and amoeba hosts in two chloraminated drinking water distribution systems. Appl Environ Microbiol 78:6285–6294. doi:10.1128/AEM.01492-12. 22752174 PMC3416603

[12] Trabelsi H, Dendana F, Sellami A, Sellami H, Cheikhrouhou F, Neji S, Makni F, Ayadi A 2012 Pathogenic free-living amoebae: epidemiology and clinical review. Pathol Biol (Paris) 60:399–405. doi:10.1016/j.patbio.2012.03.002. 22520593

[13] Olofsson J, Axelsson-Olsson D, Brudin L, Olsen B, Ellstrom P 2013 *Campylobacter jejuni* actively invades the amoeba *Acanthamoeba polyphaga* and survives within non digestive vacuoles. PLoS One 8:e78873. doi:10.1371/journal.pone.0078873. 24223169 PMC3819376

[14] Tezcan-Merdol D, Ljungstrom M, Winiecka-Krusnell J, Linder E, Engstrand L, Rhen M 2004 Uptake and replication of *Salmonella enterica* in *Acanthamoeba rhysodes*. Appl Environ Microbiol 70:3706–3714. doi:10.1128/AEM.70.6.3706-3714.2004. 15184177 PMC427739

[15] Moffat JF, Tompkins LS 1992 A quantitative model of intracellular growth of *Legionella pneumophila* in *Acanthamoeba castellanii*. Infect Immun 60:296–301. 1729191 10.1128/iai.60.1.296-301.1992 PMC257535

[16] Van der Henst C, Scrignari T, Maclachlan C, Blokesch M 2016 An intracellular replication niche for *Vibrio cholerae* in the amoeba *Acanthamoeba castellanii*. ISME J 10:897–910. doi:10.1038/ismej.2015.165. 26394005 PMC4705440

[17] Akya A, Pointon A, Thomas C 2010 *Listeria monocytogenes* does not survive ingestion by *Acanthamoeba polyphaga*. Microbiology 156:809–818. doi:10.1099/mic.0.031146-0. 19892759

[18] Akya A, Pointon A, Thomas C 2009 Viability of *Listeria monocytogenes* in co-culture with *Acanthamoeba* spp. FEMS Microbiol Ecol 70:20–29. doi:10.1111/j.1574-6941.2009.00736.x. 19645820

[19] Huws SA, Morley RJ, Jones MV, Brown MR, Smith AW 2008 Interactions of some common pathogenic bacteria with *Acanthamoeba polyphaga*. FEMS Microbiol Lett 282:258–265. doi:10.1111/j.1574-6968.2008.01123.x. 18399997

[20] Zhou X, Elmose J, Call DR 2007 Interactions between the environmental pathogen *Listeria monocytogenes* and a free-living protozoan (*Acanthamoeba castellanii*). Environ Microbiol 9:913–922. doi:10.1111/j.1462-2920.2006.01213.x. 17359263

[21] Saeed A, Abd H, Edvinsson B, Sandstrom G 2009 *Acanthamoeba castellanii* an environmental host for *Shigella dysenteriae* and *Shigella sonnei*. Arch Microbiol 191:83–88. doi:10.1007/s00203-008-0422-2. 18712360

[22] Winckler J 1974 Vital staining of lysosomes and other cell organelles of the rat with neutral red. Prog Histochem Cytochem 6:1–91. (In German.) 4142096

[23] Nemes Z, Dietz R, Luth JB, Gomba S, Hackenthal E, Gross F 1979 The pharmacological relevance of vital staining with neutral red. Experientia 35:1475–1476. doi:10.1007/BF01962793. 510488

[24] Clarke M, Maddera L 2006 Phagocyte meets prey: uptake, internalization, and killing of bacteria by *Dictyostelium amoebae*. Eur J Cell Biol 85:1001–1010. doi:10.1016/j.ejcb.2006.05.004. 16782228

[25] Maurelli AT, Blackmon B, Curtiss R III 1984 Temperature-dependent expression of virulence genes in *Shigella* species. Infect Immun 43:195–201. 6360895 10.1128/iai.43.1.195-201.1984 PMC263409

[26] Saeed A, Johansson D, Sandstrom G, Abd H 2012 Temperature depended role of *Shigella flexneri* invasion plasmid on the interaction with *Acanthamoeba castellanii*. Int J Microbiol 2012:917031. doi:10.1155/2012/917031. 22518151 PMC3299343

[27] Molmeret M, Horn M, Wagner M, Santic M, Abu Kwaik Y 2005 Amoebae as training grounds for intracellular bacterial pathogens. Appl Environ Microbiol 71:20–28. doi:10.1128/AEM.71.1.20-28.2005. 15640165 PMC544274

[28] Ram PK, Crump JA, Gupta SK, Miller MA, Mintz ED 2008 Part II. Analysis of data gaps pertaining to *Shigella* infections in low and medium human development index countries, 1984–2005. Epidemiol Infect 136:577–603. doi:10.1017/S0950268807009351. 17686195 PMC2870860

[29] Holt KE, Thieu Nga TV, Thanh DP, Vinh H, Kim DW, Vu Tra MP, Campbell JI, Hoang NV, Vinh NT, Minh PV, Thuy CT, Nga TT, Thompson C, Dung TT, Nhu NT, Vinh PV, Tuyet PT, Phuc HL, Lien NT, Phu BD, Ai NT, Tien NM, Dong N, Parry CM, Hien TT, Farrar JJ, Parkhill J, Dougan G, Thomson NR, Baker S 2013 Tracking the establishment of local endemic populations of an emergent enteric pathogen. Proc Natl Acad Sci U S A 110:17522–17527. doi:10.1073/pnas.1308632110. 24082120 PMC3808646

[30] Nuesch-Inderbinen M, Heini N, Zurfluh K, Althaus D, Hachler H, Stephan R 2016 *Shigella* antimicrobial drug resistance mechanisms, 2004–2014. Emerg Infect Dis 22:1083–1085. doi:10.3201/eid2206.152088. 27191035 PMC4880098

[31] Zhang W, Luo Y, Li J, Lin L, Ma Y, Hu C, Jin S, Ran L, Cui S 2011 Wide dissemination of multidrug-resistant *Shigella* isolates in China. J Antimicrob Chemother 66:2527–2535. doi:10.1093/jac/dkr341. 21859815

[32] Calcuttawala F, Hariharan C, Pazhani GP, Ghosh S, Ramamurthy T 2015 Activity spectrum of colicins produced by *Shigella sonnei* and genetic mechanism of colicin resistance in conspecific *S. sonnei* strains and *Escherichia coli*. Antimicrob Agents Chemother 59:152–158. doi:10.1128/AAC.04122-14. 25331695 PMC4291344

[33] Kaewklom S, Samosornsuk S, Pipatsatitpong D, Aunpad R 2013 Colicin type 7 produced by majority of *Shigella sonnei* isolated from Thai patients with diarrhoea. Braz J Microbiol 44:731–736. doi:10.1590/S1517-83822013000300010. 24516440 PMC3910181

[34] Padilla C, Lobos O, Brevis P, Abaca P, Hubert E 2006 Plasmid-mediated bacteriocin production by *Shigella flexneri* isolated from dysenteric diarrhoea and their transformation into *Escherichia coli*. Lett Appl Microbiol 42:300–303. doi:10.1111/j.1472-765X.2005.01829.x. 16478521

[35] Anderson MC, Vonaesch P, Saffarian A, Marteyn BS, Sansonetti PJ 2017 *Shigella sonnei* encodes a functional T6SS used for interbacterial competition and niche occupancy. Cell Host Microbe 21:769–776.e3. doi:10.1016/j.chom.2017.05.004. 28618272

[36] Caboni M, Pedron T, Rossi O, Goulding D, Pickard D, Citiulo F, MacLennan CA, Dougan G, Thomson NR, Saul A, Sansonetti PJ, Gerke C 2015 An O antigen capsule modulates bacterial pathogenesis in *Shigella sonnei*. PLoS Pathog 11:e1004749. doi:10.1371/journal.ppat.1004749. 25794007 PMC4368438

[37] Mahmoud RY, Stones DH, Li W, Emara M, El-Domany RA, Wang D, Wang Y, Krachler AM, Yu J 2016 The multivalent adhesion molecule SSO1327 plays a key role in *Shigella sonnei* pathogenesis. Mol Microbiol 99:658–673. doi:10.1111/mmi.13255. 26481305

[38] Parsot C, Menard R, Gounon P, Sansonetti PJ 1995 Enhanced secretion through the *Shigella flexneri* Mxi-Spa translocon leads to assembly of extracellular proteins into macromolecular structures. Mol Microbiol 16:291–300. doi:10.1111/j.1365-2958.1995.tb02301.x. 7565091

[39] Herring CD, Glasner JD, Blattner FR 2003 Gene replacement without selection: regulated suppression of amber mutations in *Escherichia coli*. Gene 311:153–163. doi:10.1016/S0378-1119(03)00585-7. 12853150

[40] Datsenko KA, Wanner BL 2000 One-step inactivation of chromosomal genes in *Escherichia coli* K-12 using PCR products. Proc Natl Acad Sci U S A 97:6640–6645. doi:10.1073/pnas.120163297. 10829079 PMC18686

[41] Liu T, Mirschberger C, Chooback L, Arana Q, Dal Sacco Z, MacWilliams H, Clarke M 2002 Altered expression of the 100 kDa subunit of the *Dictyostelium* vacuolar proton pump impairs enzyme assembly, endocytic function and cytosolic pH regulation. J Cell Sci 115:1907–1918. 11956322 10.1242/jcs.115.9.1907

[42] Mavridou DAI, Gonzalez D, Clements A, Foster KR 2016 The pUltra plasmid series: a robust and flexible tool for fluorescent labeling of Enterobacteria. Plasmid 87-88:65–71. doi:10.1016/j.plasmid.2016.09.005. 27693407

